# BioVisReport: A Markdown-based lightweight website builder for reproducible and interactive visualization of results from peer-reviewed publications

**DOI:** 10.1016/j.csbj.2022.06.009

**Published:** 2022-06-08

**Authors:** Jingcheng Yang, Yaqing Liu, Jun Shang, Yechao Huang, Ying Yu, Zhihui Li, Leming Shi, Zihan Ran

**Affiliations:** a State Key Laboratory of Genetic Engineering, Human Phenome Institute, School of Life Sciences and Shanghai Cancer Center, Fudan University, 2005 Songhu Road, Shanghai 200438, China; b Greater Bay Area Institute of Precision Medicine, 115 Jiaoxi Road, Guangzhou 511458, China; c Department of Research, Shanghai University of Medicine & Health Sciences Affiliated Zhoupu Hospital, 1500 Zhouyuan Road, Shanghai 201318, China; d Inspection and Quarantine Department, The College of Medical Technology, Shanghai University of Medicine & Health Sciences, 279 Zhouzhu Road, Shanghai 201318, China

**Keywords:** Reproducibility, Website builder, Interactive visualization, Peer-reviewed publications

## Abstract

Interactive visualization is an effective way to promote the reproducibility of results presented in biomedical publications and to facilitate additional exploration of the reported data. However, there is a lack of convenient tools that balance reproducibility with ease of use. To address this problem, we develop BioVisReport, a lightweight solution for the rapid generation of an interactive website based on a user-defined Markdown file, which acts as a text markup language without requiring users to master complex syntax and allows them to preview the results in real-time. Interactive websites generated by the tool can help readers conveniently reproduce research findings and perform further in-depth analyses beyond those reported in the original peer-reviewed publications. Currently, BioVisReport offers 17 basic types of plots for visualizing published data. In addition, the extensibility of BioVisReport supports flexible integration of user-developed Python plugins with multiple programming languages. BioVisReport is freely available at https://biovis.report/.

## Graphical abstract

**Figure:**
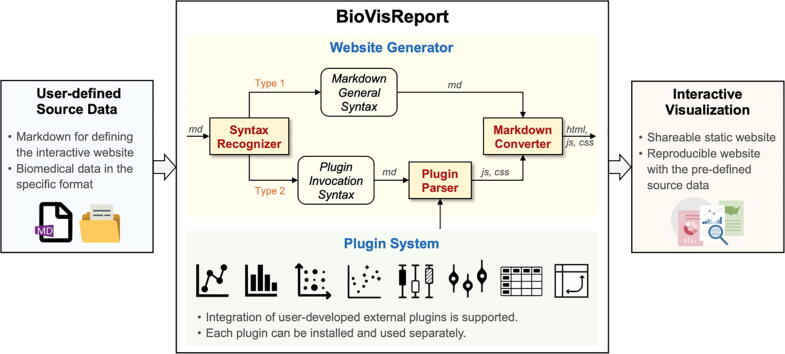

## 1 Introduction

Reproducibility of results in biomedical publications by a third party is essential but challenging [1]. The disclosure of raw data and analysis pipeline is strongly advocated to facilitate the reproducibility of results by peers [2], [3], [4]. However, it is unavoidable that there is still a large amount of research data with low accessibility, which directly limits outside researchers to repeat the analysis of publications [5], [6]. Even in the presence of data accessibility, irrational or undocumented manipulations in the analysis process, such as different software versions, parameters, threshold conditions, etc., likewise lead to irreproducibility dilemmas [7], [8].

In this context, the idea of using interactive visualisation to drive reproducibility was proposed [9]. This approach has been mainly applied to aggregated, normalized or segmented data, also known as level 3 data, such as variant calling files and quantitative expression profiles [10]. This type of data is widely used for downstream analysis and has the closest connection to the conclusions of a publication. Interactive visualization ties these data to the code for downstream analysis so that readers can analyze the data directly by point-and-click means. It allows the reader to quickly reproduce results in the publication and also perform in-depth analysis based on the interactive figures [11]. Interactive visualization differentiates itself from the traditional approach that uses a limited number of static figures to highlight the most important findings in a publication, making it possible to bridge the gap between the conclusions and the underlying data [12], [13].

Common implementations of visualizing research results interactively can be divided into two categories. One is to provide a computational notebook containing interactive visual widgets, e.g., Jupyter Notebooks with ipywidgets [14], which essentially provides readers with code and data for re-analysis. However, improper coding by the authors may lead to a high degree of irreproducibility [15], [16]. Another more effective way is to provide a stand-alone online website that can be easily accessed through a URL. However, this approach not only requires a high level of programming skills and web development capabilities for the authors but is also time-consuming and labor-intensive.

Here, we developed BioVisReport, a lightweight website builder for interactive visualization reports, to facilitate the implementation of the FAIR (Findability, Accessibility, Interoperability, and Reusability) principle in biomedical publications [17]. Users can generate a website with this tool by writing a Markdown file, a text markup language with the feature of writing what you get and previewing the results in real time [18]. Users can format the text content with some simple markups, such as adding different numbers of “#” at the beginning of the lines to set them as different levels of headers. Researchers can attach the website address to their publication, allowing readers to use it to reproduce the original results and dig further into the data.

## 2 Materials & methods

**Fig. 1:**
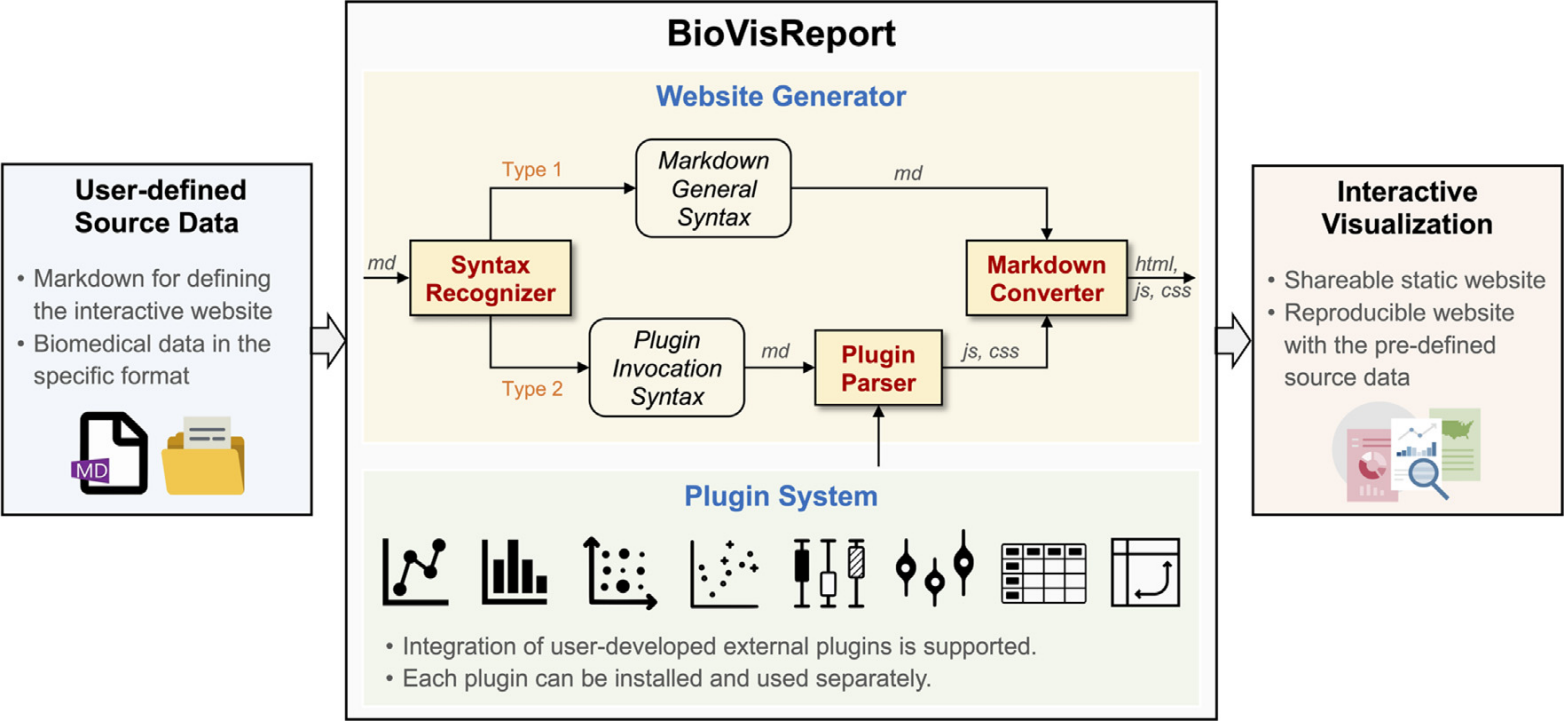
The architecture of BioVisReport. The Website Generator can recognize the syntax of the Markdown file and divide the commands into two categories: (1) for the Markdown general syntax, it will be converted into elements of the website, i.e., *html*, *js* and *css*; (2) for the commands invocating plugins, a special parser is designed for generating *js* and *css* elements, which will be passed to the Markdown converter to participate in generating the final website. In addition, the Plugin System provides plugins capable of making interactive plots.

BioVisReport invokes specified plugins and biomedical data based on a user-defined Markdown file, and then generates an interactive website. This work is achieved through two components of BioVisReport, i.e., the website generator and the plugin system (Fig. 1).

### 2.1 Website generator

Website generator is built using Python and additional libraries including Jinja2 [19], Markdown [18], Tornado [20], Mkdocs [21], and livereload [22]. The generation of an interactive website requires the user to prepare data, set the website format and specify plot plugins. In BioVisReport, all the above tasks can be defined in a Markdown file. A syntax recognizer was developed to parse the plugin invocation syntax in the Markdown file and then execute these commands. Each plugin may receive one or more input files. The files can be located on a local disk or remotely, and can be in a tabular data file format (CSV or TSV), Rdata, or other formats. All data files are first downloaded or cached and then loaded using the built-in Python or R libraries. A Markdown converter receives the elements generated by the syntax recognizer and, at the same time, converts other commands in the Markdown file into the corresponding HTML statements to achieve the layout effect expected by the user.

### 2.2 Plugin system

The plugin system contains a variety of mutually independent plot plugins that can be invoked by the website generator. Our strategy is to develop separate plugins for each type of graph to achieve compatibility among multiple programming languages and a high level of extensibility. Each plugin has been encapsulated as a Python package and can be installed separately. BioVisReport recognizes Python packages through an API interface. This design strategy allows users with programming skills to develop customized plugins that are not yet supported in BioVisReport. All currently available plugins were developed using Python, JavaScript, R and additional libraries including Plotly [23], Dplyr [24], Shiny [25], ggplot2 [26], WebDataRocks [27], etc.

In addition, BioVisReport is designed to accommodate non-expert users by supporting both development and production modes, improving usability through faster feedback. In development mode, the instance follows the principle of immediate feedback, and it will generate the website and embed a Markdown editor into the website. Through the Markdown editor, the user can modify the Markdown file and trigger the instance to respond to modifications in real-time.

## 3 Results

### 3.1 Overview of BioVisReport

BioVisReport has been implemented as a Conda package (biovis-report) in development mode for easy installation and as a Docker image (biovis-report-viewer) in production mode for fast deployment and migration. The installation, usage manual and application examples are available at https://biovis.report/.

BioVisReport has integrated 17 basic and commonly used plugins (Table 1). In addition to the basic data table, pivot tables that allow users to filter and combine calculations to produce timely statistical charts are provided. Besides, these plugins can be used to draw pie chart, bar plot, box plot, correlation plot, density plot, group-box plot, heatmap, line plot, rocket plot, scatter plot, stack bar plot, upset plot, and violin plot. Finally, MultiQC [28] reports can be integrated, which means that users can quickly develop more comprehensive and richer reports based on the power of MultiQC, thus greatly extending the scope of applications.

**Table 1:** Summary of interactive plot plugins currently available within BioVisReport.

No.	Interactive Plugin	Usage
1	Data table based on DataTables	@datatable-js (dataUrl)
2	Pivot table based on WebDataRocks and Highcharts	@pivot-table-js (dataUrl, enableLocal)
3	Pie chart based on Echarts	@pie-chart-js (dataUrl, group, subgroup, value, title, radius, chartName, legendOrient, legendPosition, selectedMode)
4	Table based on Tabulator	@tabulator-js (dataUrl)
5	Bar plot from a Shiny app	@barplot-r (dataFile, dataType, title, xAxis, xTitle, yAxis, yTitle, colorAttr, shapeAttr, xLog10, enableSE, showpanel, subtitle, text, queryURL)
6	Box plot from a Shiny app	@boxplot-r (dataFile, dataType, title, xAxis, xTitle, xAngle, yAxis, yTitle, colorAttr)
7	Correlation plot from a Shiny app	@corrplot-r (dataFile, dataType, corrVars, title, xTitle, xAngle, yTitle, corrMethod, corrType, hcOrder, hcMethod, showLab, showPanel)
8	Density plot from a Shiny app	@density-plot-r (dataFile, dataType, title, xAxis, xTitle, colorAttr, subtitle, text, fillEnable)
9	Group-box plot from a Shiny app	@grouped-boxplot-r (dataFile, dataType, title, xAxis, xTitle, yAxis, yTitle, colorAttr, labelAttr, legendTitle, subtitle, text)
10	Heatmap from a Shiny app	@heatmap-d3-r (dataFile, dataType, rowv, colv, distfun, hclustfun, showpanel, scale, labRow, labCol, colNameLst)
11	Line plot from a Shiny app	@lineplot-r (dataFile, dataType, title, xAxis, xTitle, yAxis, yTitle, colorAttr, shapeAttr, xLog10, enableSE, showpanel, subtitle, text, queryURL)
12	Rocket plot from a Shiny app	@rocket-plot-r (dataFile, dataType, title, subtitle, xAxis, xTitle, yAxis, yTitle, xAngle, labelAttr, method, pointAlpha, pointSize, text)
13	Scatter plot from a Shiny app	@scatter-plot-r (dataFile, dataType, xAxis, yAxis, sizeAttr, nameAttr, colorAttr, labelAttr, showpanel)
14	Stack bar plot from a Shiny app	@stack-barplot-r (dataFile, dataType, title, xAxis, xTitle, yAxis, yTitle, xAngle, labelAttr, subtitle, text, barPos, smartColor)
15	Upset plot from a Shiny app	@upset-r (dataFile, dataType, title, showEmptyInterSec, showBarNumbers, setSort, nIntersects, assignmentType, subtitle, text, showpanel)
16	Violin plot from a Shiny app	@violin-plot-r (dataFile, dataType, title, xAxis, xTitle, yAxis, yTitle, xAngle, colorAttr, subtitle, text)
17	MultiQC	@multiqc-py (analysisDir)

### 3.2 Usage

**Fig. 2:**
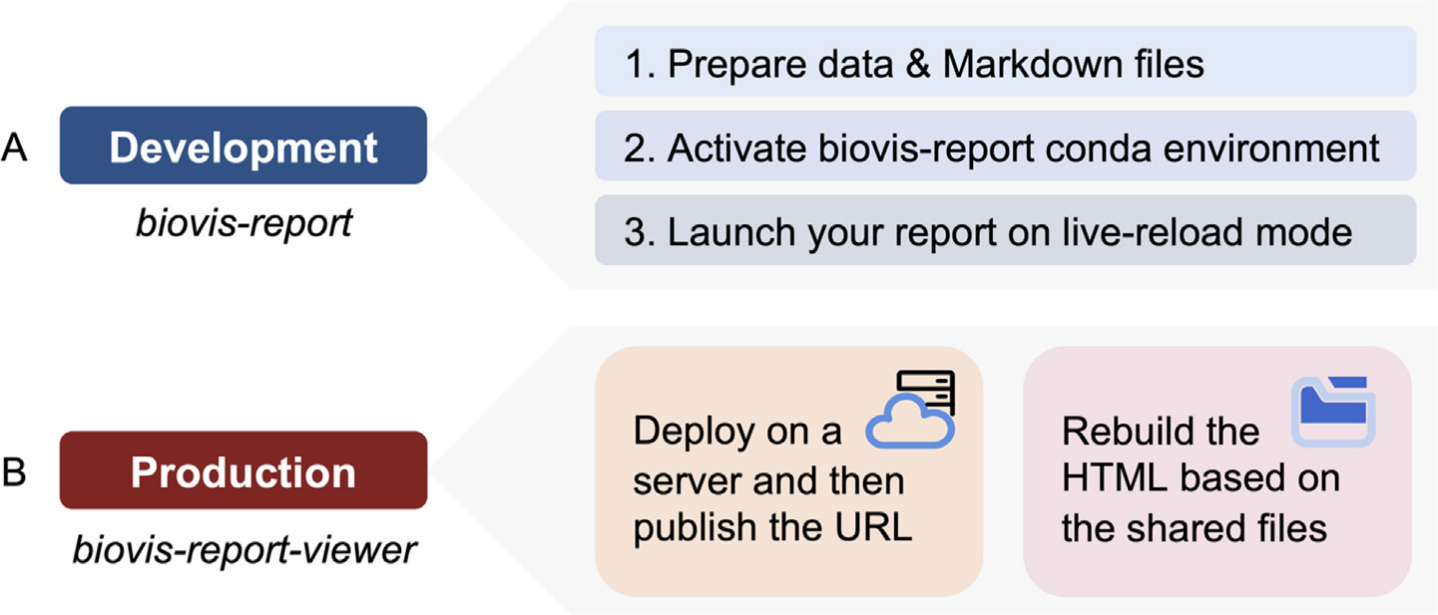
Development and production modes of BioVisReport. (A) In development mode, users prepare the data and Markdown files, and then develop the report in a live-reload state of biovis-report. (B) In production mode, the interactive reports can be published and deployed to the server, or can be reproduced with the original data using biovis-report-viewer.

During the development stage, the design and content of the reports often need to be constantly adjusted. In this case, we provide biovis-report, a Conda package that contains all the dependent plugins and supports live-reloading (Fig. 2A). Users can immediately generate an interactive report based on Markdown files and simple terminal commands, with changes to Markdown reacting to the report in real time. Report generation with biovis-report consists the following steps, including preparing data, designing the Markdown templates, activating Conda environment to run BioVisReport and then obtain the HTML file.

In production mode, biovis-report-viewer is suitable for formal releasing and sharing after the final report has been generated (Fig. 2B). Here, we recommend two approaches to apply the interactive reports generated by BioVisReport to publications. On the one hand, authors can deploy the locally generated reports on a server, i.e., transform a temporary HTML into a URL that can be stably accessed. In particular, for purely static websites entirely based on JavaScript plugins, server deployment is even not required, only the HTML file is stored on a publicly accessible cloud drive. Then authors can attach the website address to the publication. On the other hand, authors can attach the Markdown file with the corresponding data to their publications and inform the readers to install the docker image biovis-report-viewer. The interactive website can be reproduced on the readers’ end with just one line of command.

### 3.3 Example of interactive visualization report

To better demonstrate the capabilities of BioVisReport, we provide an example based on the genomic and transcriptomic profiling of the Chinese triple-negative breast cancer (TNBC) cohort [29]. The details are described below and the report can be viewed through the Video S1.

This report contains a total of four interfaces, i.e., home page, genomic alterations, gene expression, metadata and clinical information. Among them, the home page contains the abstract of the Chinese TNBC report. It does not invoke any interactive plugins, but is converted directly from the Markdown file. The theme of the report is set via command line parameters and the graphical abstract in the center of the page is stored in the working directory in advance. Other interfaces invoked interactive plugins, and the Markdown information and the corresponding results are as follows.

#### 3.3.1 Genomic Alternations

**Fig. 3:**
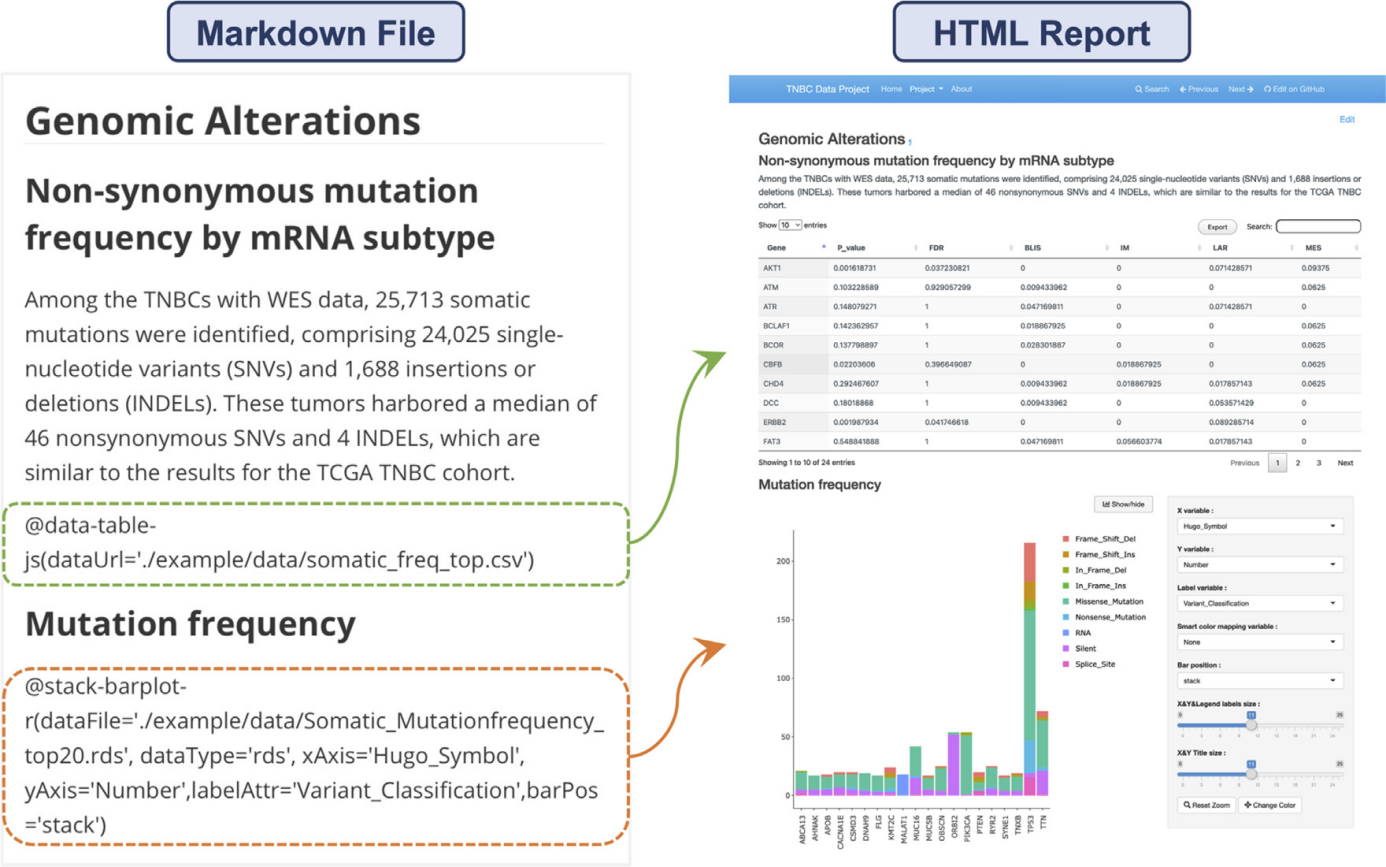
The “Genomic Alternations” interface shows the variants through data table and bar chart by mRNA subtype, variant classification and gene symbol. The commands to invoke plugins in the Markdown file and the final outcome are shown by the arrows.

The “Genomic Alternations” tab summarizes somatic SNPs and INDELs in Chinese TNBC with WES data at the beginning (Fig. 3). Then, the data table summarizes the non-synonymous variant frequency by mRNA subtype, along with a *p*-value and an FDR value. The bar chart displays the gene variant frequency stacked by variant classification. Users can change variables (x or y variable, smart color mapping variable, bar position, x and y legend labels or font size of the title, etc.) with the dropdowns and scrollbars in the sidebar panel.

#### 3.3.2 Gene expression

**Fig. 4:**
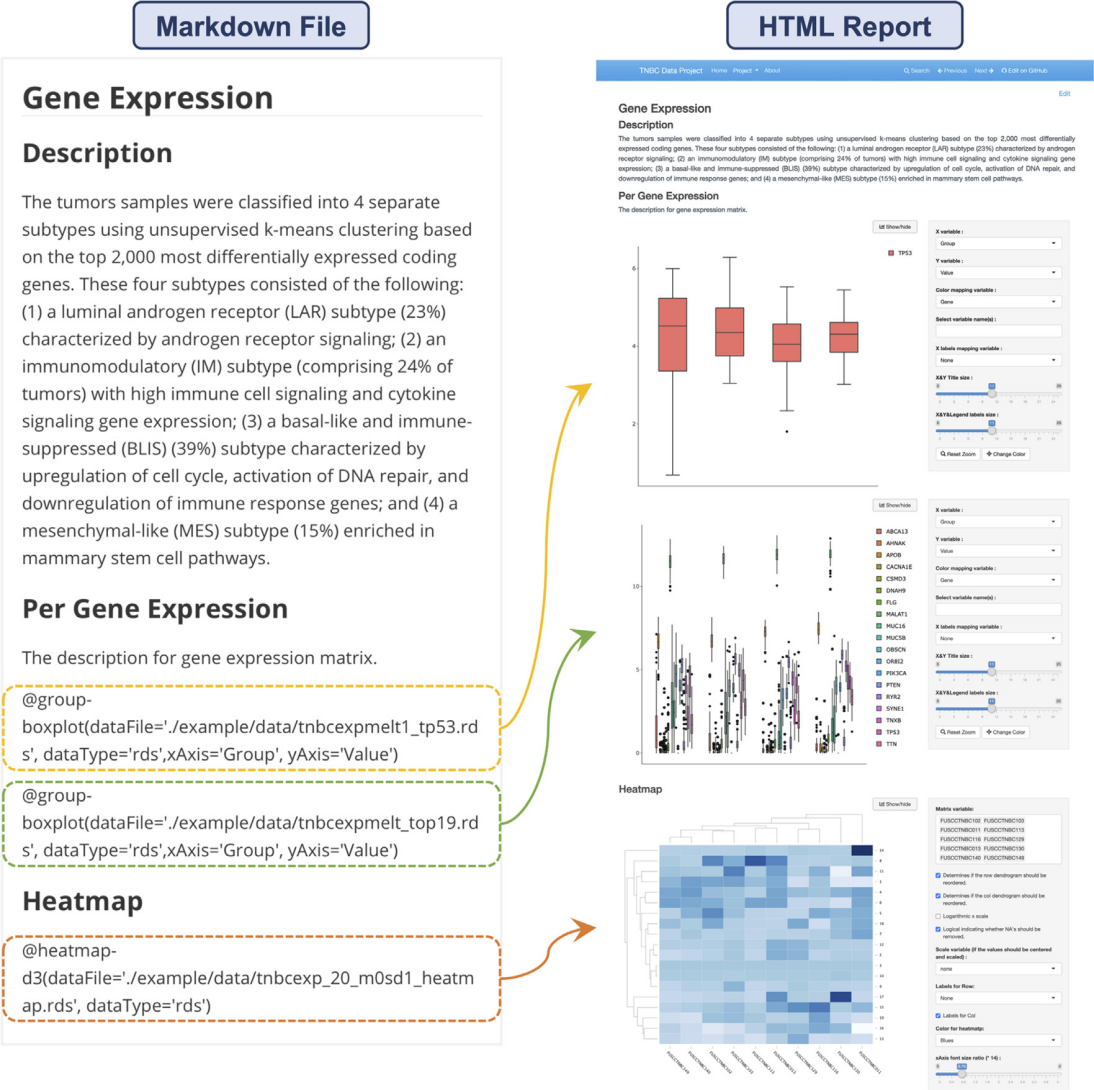
The “Gene Expression” interface displays data in the form of heatmap and box plot. The commands to invoke plugins in the Markdown file and the final outcome are shown by the arrows.

The “Gene Expression” tab integrates heatmap and box plot (Fig. 4). The heatmap allows users to visualize gene-expression data for specified patients selected through the checkbox. This panel also enables users to determine whether to center and scale the value, reorder the dendrogram in row or column, take the x scale logarithm and remove NA values. The box plot shows distribution of the gene-expression levels and users can choose genes they are interested in by clicking on the legend. And the second box plot displays the mapping ratio of two batches of samples.

#### 3.3.3 Meta data and clinical information

**Fig. 5:**
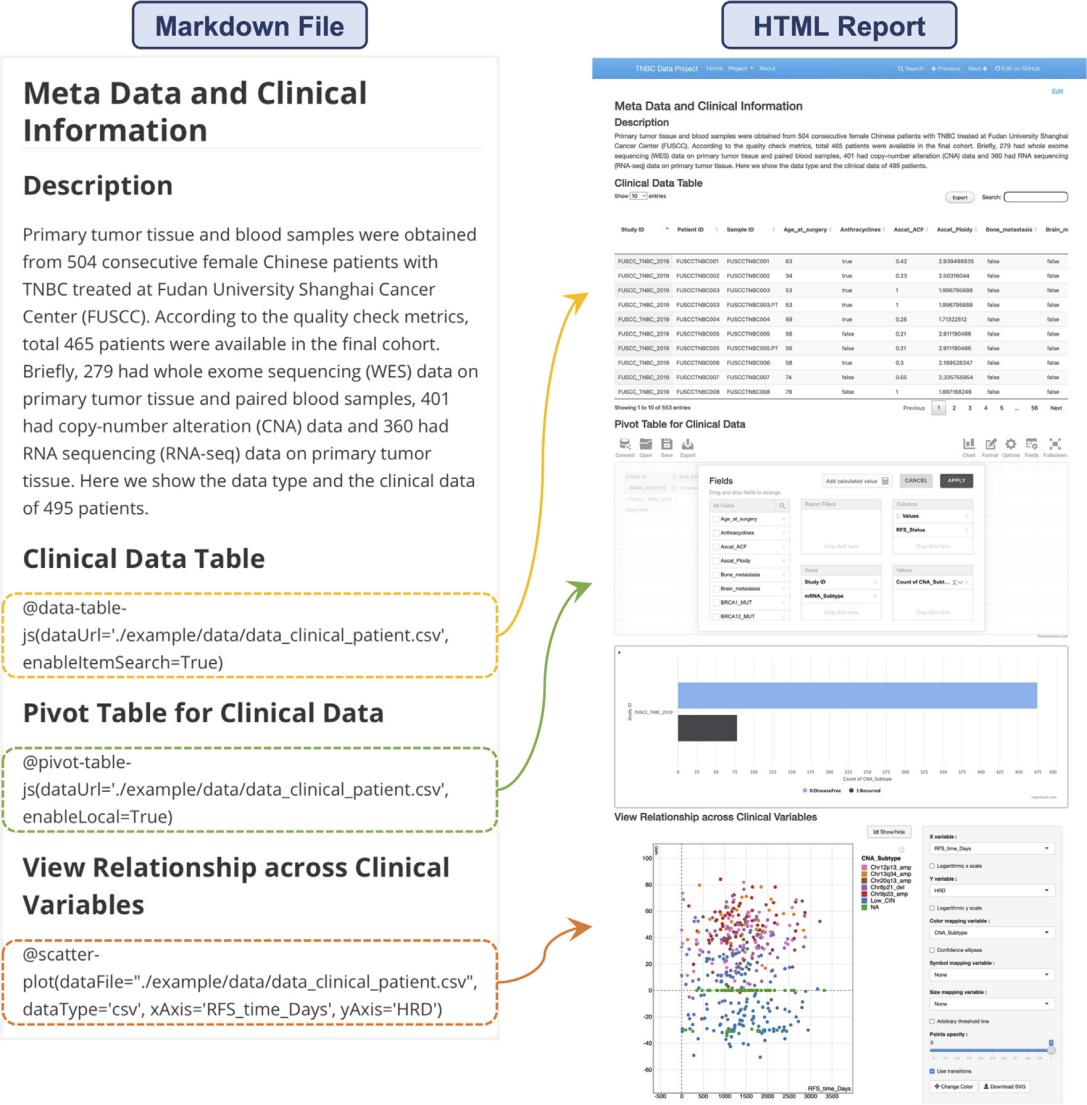
The “Meta Data and Clinical Information” interface integrates data table, pivot table and scatter plot which allows users to view relationships across clinical variables. The commands to invoke plugins in the Markdown file and the final outcome are shown by the arrows.

The “Meta Data and Clinical Information” tab shows the data type and the clinical data of 495 patients in this cohort (Fig. 5). The data table, which is used to show the clinical data, allows users to query any variable and the pivot table can be used to analyze and organize the complex data. Finally, a scatter plot shows the relationship across clinical variables. These custom features, such as color, symbol or size of mapping variables, arbitrary threshold lines, and confidence ellipses, are provided for users to mine in detail of the correlation between variables.

## 4 Conclusions

BioVisReport is a lightweight solution for quickly generating an interactive website for reproducing figures associated with a publication. It currently offers 17 basic plugins that can cover the needs of common scenarios of data visualization. Due to the excellent scalability of BioVisReport's system architecture, it allows quick extensions with encapsulating user-defined tools. For example, the integration of a visualization method of a new omic-type data only requires the encapsulation of the existing code as a new plugin. In addition, in order to simplify the development of interactive websites for data exploration and visualization analysis tasks, we have worked on two fronts. On the one hand, we adopted easy-to-use and instantly visible Markdown files in the website design process. On the other hand, during the website modification and optimization process, we provided the development mode so that when users modify data or Markdown files, the ongoing BioVisReport instance can respond to the changes in real-time.

Importantly, BioVisReport has been used by external researchers to build their data portal, further demonstrating the utility of our tool in biomedical peer-reviewed publications [30]. However, more simplified usage, extensive documentation and a wide range of plugins are required for further promotion and adoption. For example, in the current version it is difficult for non-expert users who do not know the Markdown language and do not have an environment, e.g., Anaconda, to carry out its development. We plan to address the issue in a subsequent development of a dashboard that works in a drag-and-drop style. Users can drag the elements and then the Markdown file will be generated automatically. Based on this, we also plan to integrate and provide more visualization plugins.

Overall, the model of applying interactive visualization reporting advocated by BioVisReport, makes the data behind the conclusions less limited by the graphical presentation, thus helping readers to better understand the hypotheses and results conveyed in publications. At the same time, it also helps to identify problems of publication bias and selective reporting, which will improve the accuracy of results generation and interpretation. We believe that BioVisReport will be an effective tool in peer-reviewed publications and even in daily academic communication, thus contributing to the reproducibility of biomedical discoveries.

### CRediT authorship contribution statement

**Jingcheng Yang:** Conceptualization, Methodology, Software, Writing – review & editing, Validation, Resources. **Yaqing Liu:** Conceptualization, Writing – original draft, Resources, Visualization. **Jun Shang:** Conceptualization, Software, Resources. **Yechao Huang:** Software, Resources. **Ying Yu:** Resources, Validation. **Zhihui Li:** Resources, Validation. **Leming Shi:** Conceptualization, Writing – review & editing, Validation. **Zihan Ran:** Project administration, Funding acquisition, Writing – review & editing.

## Declaration of Competing Interest

The authors declare that they have no known competing financial interests or personal relationships that could have appeared to influence the work reported in this paper.
